# The Targeting of MRE11 or RAD51 Sensitizes Colorectal Cancer Stem Cells to CHK1 Inhibition

**DOI:** 10.3390/cancers13081957

**Published:** 2021-04-19

**Authors:** Luca Mattiello, Sara Soliman Abdel Rehim, Martina Musella, Antonella Sistigu, Andrea Guarracino, Sara Vitale, Francesca Corradi, Claudia Galassi, Francesca Sperati, Gwenola Manic, Ruggero De Maria, Ilio Vitale

**Affiliations:** 1 Italian Institute for Genomic Medicine (IIGM), c/o IRCSS, 10060 Candiolo, Italy; mattiello.borsisti@iigm.it (L.M.); soliman.borsisti@iigm.it (S.S.A.R.); andrea.guarracino@uniroma2.it (A.G.); 2 Candiolo Cancer Institute, FPO—IRCCS, 10060 Candiolo, Italy; 3 Department of Biology, University of Rome “Tor Vergata”, 00133 Rome, Italy; francesca.corradi89@gmail.com; 4 Dipartimento di Medicina e Chirurgia Traslazionale, Università Cattolica del Sacro Cuore, 00168 Rome, Italy; martina.musella@unicatt.it (M.M.); antonella.sistigu@unicatt.it (A.S.); sara.vitale@unicatt.it (S.V.); claudia.galassi@unicatt.it (C.G.); 5 UOSD Tumor Immunology and Immunotherapy Unit, IRCCS Regina Elena National Cancer Institute, 00144 Rome, Italy; 6 UOSD Biostatistics, Bioinformatics and Clinical Trial Center, San Gallicano Dermatological Institute IRCCS, 00144 Rome, Italy; francesca.sperati@ifo.gov.it; 7 Fondazione Policlinico Universitario “A. Gemelli”—IRCCS, 00168 Rome, Italy

**Keywords:** colorectal cancer, chromosomal instability, DNA damage, targeted therapy, tumor-initiating cells

## Abstract

**Simple Summary:**

The ATR-CHK1 axis of the DNA damage response is crucial for the survival of most colorectal cancer stem cells (CRC-SCs), but a significant fraction of primary CRC-SCs either is resistant to ATR or CHK1 inhibitors or survives the abrogation of the ATR-CHK1 cascade despite an initial response. Here, we demonstrate that the targeting of RAD51 or MRE11 improves the sensitivity of primary CRC-SCs to the CHK1/2 inhibitor prexasertib by sequentially inducing replication stress, the abrogation of cell cycle checkpoints, and the emergence of mitotic defects. This results in the induction of mitotic catastrophe and CRC-SC killing via a caspase-dependent apoptosis.

**Abstract:**

Cancer stem cells (CSCs) drive not only tumor initiation and expansion, but also therapeutic resistance and tumor relapse. Therefore, CSC eradication is required for effective cancer therapy. In preclinical models, CSCs demonstrated high capability to tolerate even extensive genotoxic stress, including replication stress, because they are endowed with a very robust DNA damage response (DDR). This favors the survival of DNA-damaged CSCs instead of their inhibition via apoptosis or senescence. The DDR represents a unique CSC vulnerability, but the abrogation of the DDR through the inhibition of the ATR-CHK1 axis is effective only against some subtypes of CSCs, and resistance often emerges. Here, we analyzed the impact of druggable DDR players in the response of patient-derived colorectal CSCs (CRC-SCs) to CHK1/2 inhibitor prexasertib, identifying RAD51 and MRE11 as sensitizing targets enhancing prexasertib efficacy. We showed that combined inhibition of RAD51 and CHK1 (via B02+prexasertib) or MRE11 and CHK1 (via mirin+prexasertib) kills CSCs by affecting multiple genoprotective processes. In more detail, these two prexasertib-based regimens promote CSC eradication through a sequential mechanism involving the induction of elevated replication stress in a context in which cell cycle checkpoints usually activated during the replication stress response are abrogated. This leads to uncontrolled proliferation and premature entry into mitosis of replication-stressed cells, followed by the induction of mitotic catastrophe. CRC-SCs subjected to RAD51+CHK1 inhibitors or MRE11+CHK1 inhibitors are eventually eliminated, and CRC-SC tumorspheres inhibited or disaggregated, via a caspase-dependent apoptosis. These results support further clinical development of these prexasertib-based regimens in colorectal cancer patients.

## 1. Introduction

Robust experimental evidence indicates that human tumors often exhibit a hierarchical organization, whose apex is occupied by a subpopulation of cancer cells known as cancer stem cells (CSCs) because of the ability to self-renew and generate a progeny with different degree of differentiation (reviewed in [1]). CSCs have been identified and prospectively isolated from colorectal and other cancers, where they are believed to promote tumor development and to contribute to disease expansion, evolution and dissemination [2,3,4,5,6]; for these reasons, they are also known as tumor-initiating cells or tumor-propagating cells. In addition, CSCs have been shown to act as a main source of tumor heterogeneity [7], which is in turn linked to dismal prognosis and therapy resistance, as well as of tumor relapse [1,8]. At least in part, these features are linked to the relative low proneness of CSCs to undergo regulated cell death under stress conditions. In particular, in preclinical patient-derived models, CSCs have demonstrated elevated resistance to DNA damages, making them able to tolerate constitutive replication stress–defined as the slowing or stalling of replication fork progression and/or DNA synthesis [9]–or survive conventional genotoxic agents, including ionizing radiation and chemotherapeutics (reviewed in [10]). 

The current view is that the resistance of CSCs derives from the orientation of the DNA damage response (DDR) towards cytoprotection. The DDR is a multipronged mechanism specifically activated in cells experiencing DNA lesions, operating through a two-step sensing-signal transduction cascade. This culminates either in the activation of cytoprotective signaling modules favoring the repair of or tolerance to DNA lesions, or in the activation of cytotoxic signaling modules leading to proliferation arrest or demise of irreversibly damaged cells upon induction of cell senescence or regulated cell death [10,11]. The activation of these pathways depends on the severity of the insults as well as on the efficiency of the machineries responsible for sensing, repairing, or tolerating DNA lesions [10]. Other crucial factors orienting the DDR towards cytosurvival or cytotoxicity encompass the proficiency, robustness, and activation kinetics of cytoprotective versus cytosurvival pathways. Collectively, these features dictate the outcome (i.e., survival or death) of DNA-damaged cells. 

There is evidence that CSCs survive extensive DNA damage because of their capability not only to tolerate and repair DNA lesion, but also to detoxify reactive oxygen species and/or extrude DNA-damaging drugs [12,13,14]. Other factors tipping the balance toward the survival of CSC experiencing extensive DNA lesions include: (i) limited activation (or silencing) of cytotoxic mechanisms, which is due to the intrinsic deregulation of apoptotic pathways and evasion of regulated cell death or senescence [15,16], together with (ii) high proneness to activate cytosurvival cascades, originating from the constitutive activation of the DDR signaling [17,18,19]. In particular, a basal overactivaton of the ataxia telangiectasia mutated serine/threonine kinase (ATM)-checkpoint kinase 2 (CHEK2, best known as CHK2) axis, which is primarily activated by double-strand breaks (DSBs), or the ataxia telangiectasia mutated and Rad3 related serine/threonine kinase (ATR)-checkpoint kinase 1 (CHEK1, best known as CHK1) axis, which is primarily activated by long stretches of single-stranded DNA (ssDNA) adjacent to double-stranded (ds)/ssDNA junctions during replication stress, has been observed in multiple experimental models of CSC enrichment, including patient-derived models [20,21,22].

Importantly, the presence of a robust DDR constitutes a vulnerability of CSCs. On the one hand, the targeting of these DDR kinases in combination with DNA damaging agents de facto silences the cytosurvival modules of the DDR, thus reestablishing tumor sensitivity to DNA damage. On the other hand, CSCs can be particularly dependent on DDR kinase(s) for survival even in the absence of exogenous sources of DNA damage, making them targetable by the abrogation of the DDR signaling. Therefore, the DDR can be harnessed to revert the intrinsic capacity of CSCs to evade apoptosis. Accordingly, inhibitors of the DDR kinase ATM, ATR and CHK1 are reported to kill CSCs when administered either alone [20,23] or in combination with other DNA damaging agents to which CSCs were formerly resistant [17,19,24,25]. 

However, one emerging concept is that the response of CSCs to inhibitors of DDR kinases is heterogeneous, restricting the therapeutic use of these drugs to specific subtypes. Although most colorectal CSCs (CRC-SCs) are sensitive to inhibitors of the ATR-CHK1 cascade ultimately succumbing via replication catastrophe subsequent to the induction of intolerable or lethal levels of replication stress, a significant fraction of CSCs survives this regimen [20]. As a further limitation of these strategies, we recently revealed a mechanism of resistance to ATR-CHK1 inhibitors in CRC-SCs based on upregulation of poly(ADP-ribose) polymerase 1 (PARP1) [26], a DDR player with pleiotropic roles in DNA damage repair, the response to replication stress, and regulated cell death [27]. 

Based on these considerations, in this study, we investigated the impact of relevant druggable DDR players on the survival of patient-derived CRC-SCs, identifying MRE11 homolog, double-strand break repair nuclease (MRE11) and RAD51 recombinase (RAD51) as targets for sensitizing CRC-SCs to CHK1 inhibitors.

## 2. Results

### 2.1. Identification of RAD51 and MRE11 as Sensitizing Targets to Enhance the Efficacy of the CHK1 Inhibitor Prexasertib in CRC-SCs

**Figure 1:**
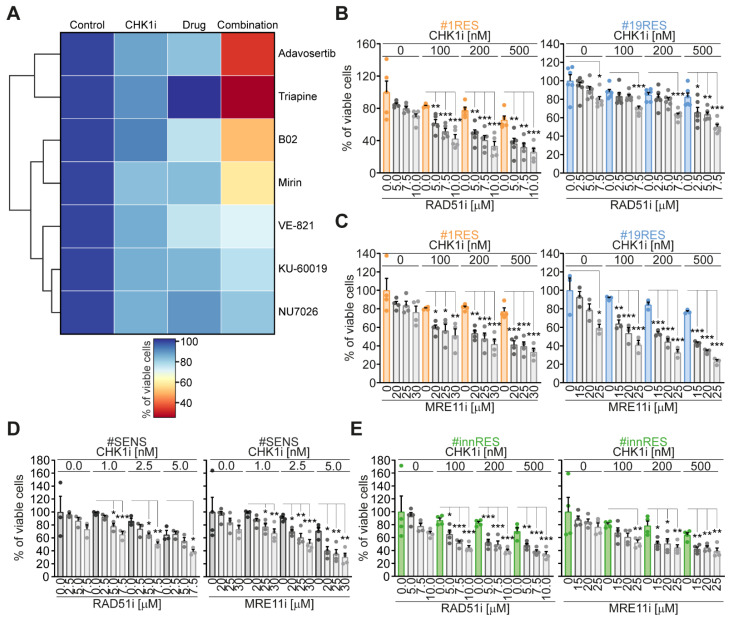
Identification of RAD51 and MRE11 inhibitors as prexasertib-sensitizing agents in CRC-SCs. (**A**) CRC-SCs previously characterized as resistant to ATR-CHK1 abrogation (RES-CRC-SCs) were left untreated or treated for 72 h with the CHK1/2 inhibitor prexasertib (CHK1i), and/or a set of modulators of the DNA damage response (DDR) with known prexasertib-sensitizing effect (i.e., adavosertib and triapine) or with unknown impact on prexasertib CSC toxicity (i.e., B02, KU-60019, mirin, NU7026, and VE-821), as indicated. Cell proliferation and viability were assessed by CellTiter-Glo^®^ assay. The heatmap shows prexasertib-sensitizing effects of DDR modulators, with values corresponding to the percentage of viable cells upon normalization on control conditions. Data are means of three independent experiments, with values reported in Supplementary Table S1. The heatmap and clusterization were generated with Python. (**B**,**C**) Cell proliferation/viability (assessed by CellTiter-Glo^®^ assay) of distinct RES-CRC-SCs left untreated or exposed for 96 h to CHK1i alone or in combination with RAD51i (B02) (**B**) or MRE11i (mirin) (**C**), as indicated. Results are means±SEM and individual data points of six (RAD51i-treated #19RES), five (RAD51i-treated #1RES), four (MRE11i-treated #1RES), or three (MRE11i-treated #19RES) independent experiments. * *p* < 0.05, ** *p* < 0.01, *** *p* < 0.001 (one-way ANOVA and Bonferroni or Dunnett’s T3 post-hoc test) as reported. (**D**,**E**) Cell proliferation/viability (evaluated by CellTiter-Glo^®^ assay) of representative CRC-SCs sensitive to CHK1i (SENS-CRC-SCs) (**D**) or intrinsically resistant to CHK1i (innRES-CRC-SCs) (**E**) left untreated or subjected for 96 h to CHK1i alone or in combination with MRE11i or RAD51i, as indicated. Results are means ± SEM and individual data points of four (MRE11i-treated SENS-CRC-SCs and #innRES) or three (RAD511i-treated SENS-CRC-SCs) independent experiments. * *p* < 0.05, ** *p* < 0.01, *** *p* < 0.001 (one-way ANOVA and Bonferroni or Dunnett’s T3 post-hoc test) as reported. Associated supplementary table: Supplementary Table S1.

We recently established distinct pairs of primary CRC-SCs sensitive (SENS) and resistant (RES) to pharmacological inhibitors of ATR and CHK1 kinases [26], the principal transducers of the cellular response to replication stress. From this panel, we selected two pairs of CRC-SCs: #1SENS/#1RES and #19SENS/#19RES, and used them to identify novel actionable targets improving the sensitivity and/or reverting the resistance to CHK1 inhibitors. We first analyzed the impact of crucial druggable DDR players on RES-CRC-SC survival. To this aim, RES-CRC-SCs were treated or not with the CHK1 inhibitor prexasertib together with inhibitors of ATR (VE-821), ATM (KU-60019), DNA-PK (NU-7026), MRE11 (mirin), and RAD51 (B02). After (co)treatments, cells were assessed for their proliferation/survival with a CellTiter-Glo^®^ Luminescent Cell Viability assay. As internal comparison, RES-CRC-SCs were exposed to two representative pharmacological agents we previously demonstrated to potently sensitize to ATR/CHK1 inhibitors: triapine and adavosertib, which inhibit, respectively, the ribonucleotide reductase regulatory subunit M2 (RRM2) and WEE1 G2 checkpoint kinase (WEE1). This analysis led to the identification of the inhibitors of MRE11 (mirin) and of RAD51 (B02) as novel sensitizers of CRC-SCs to prexasertib (Figure 1A, Table S1). As opposed to mirin and B02, pharmacological inhibitors of the DDR kinases ATM, ATR and DNA-PK were ineffective or mildly effective in sensitizing CRC-SCs to prexasertib (Figure 1A, Table S1). This result rules out redundant or parallel roles of DDR kinases in the replication stress of CSCs, also excluding a potential contribution of ATR in resolving non-stringent RS independently of CHK1, as previously reported [28].

In following dose-response studies, we confirmed that the inhibition of RAD51 or MRE11 mildly affected the survival of RES-CRC-SCs when administered alone (Figure 1B,C). On the contrary, both mirin and B02 sensitized RES-CRC-SCs to CHK1 inhibitors (Figure 1B,C), thus validating our previous result. Along similar lines, B02 or mirin administered as monotherapies had minimal effect also on the survival of SENS-CRC-SCs (Figure 1D) and on that of CRC-SCs of our panel intrinsically resistant to CHK1 inhibitors (innRES-CRC-SCs) (Figure 1E). However, these agents increased the responsiveness of all these cells to CHK1 inhibition. Indeed, the inhibition of RAD51 or of MRE11 promoted sensitization of SENS-CRC-SCs (Figure 1D) and of innRES-CRC-SCs (Figure 1E) to sublethal doses of prexasertib. In conclusion, these findings demonstrate that the targeting of MRE11 or RAD51 sensitizes CRC-SCs to the inhibition of CHK1.

### 2.2. The Targeting of RAD51 or MRE11 Sensitize to CHK1 Inhibitors by Boosting Replication Stress 

**Figure 2:**
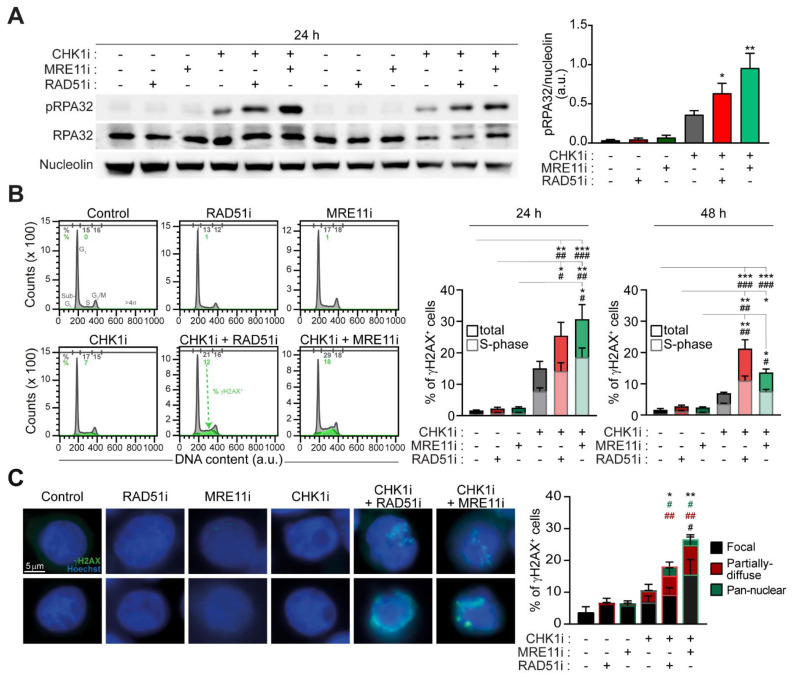
Combined inhibition of CHK1 and MRE11 or CHK1 and RAD51 induces replication stress in prexasertib-resistant CRC-SCs. (**A**) Western-blot analysis of representative RES-CRC-SCs left untreated or administrated for 24 h with prexasertib (CHK1i), either alone or in combination with MRE11i (mirin) or RAD51i (B02), and then stained with antibodies recognizing phospho(p)RPA32 (S4/S8) and RPA32 (as markers of replication stress, RS) and nucleolin (to ensure equal lane loading). One representative western-blot and the quantification of the ratio pRPA32/nucleolin are shown (see also Supplementary Figure S1). Results are expressed as means±SEM and individual data points of five independent experiments. * *p* < 0.05, ** *p* < 0.01, *** *p* < 0.001 (Kruskal-Wallis test and Dunn’s post-hoc test) compared to untreated conditions. (**B**) Flow cytometry analysis in representative RES-CRC-SCs left untreated or treated with CHK1i, either alone or combined with MRE11i or RAD51i, and then stained with a DNA intercalant (DAPI) together with an anti-γH2AX antibody. Cell cycle profiles and quantitative data (means ± SEM; six independent experiments at 24 h and seven independent experiments at 48 h) are reported. In cell cycle profiles, cells positive for γH2AX in S-phase are in green. Numbers indicate the percentage of corresponding events. In the histograms, the percentage of γH2AX^+^ cells in all cell cycle phases (total) are in dark color, while the percentage of γH2AX^+^ cells in S-phase are in pale color. * *p* < 0.05, ** *p* < 0.01, *** *p* < 0.001 (Kruskal-Wallis test and Dunn’s post-hoc test), as indicated (for γH2AX^+^ cells in all cell cycle phases). ^#^ *p* < 0.05, ^##^ *p* < 0.01, ^###^ *p* < 0.001 (Kruskal-Wallis test and Dunn’s post-hoc test), as indicated (for γH2AX^+^ cells in S-phase). (**C**) Immunofluorescence analysis in representative RES-CRC-SCs left untreated or exposed for 24 h to CHK1i, either alone or in combination with MRE11i or RAD51i, and then strained with an antibody recognizing γH2AX. Representative images and quantification of percentages of γH2AX^+^ cells presenting “focal”, “partially diffuse” or “pan-nuclear” γH2AX positivity are shown. For more information about the category of γH2AX positivity, see Materials and Methods. Data are expressed as means±SEM and individual data points of five independent experiments. * *p* < 0.05, ** *p* < 0.01, *** *p* < 0.001 (Kruskal-Wallis test and Dunn’s post-hoc test) compared to untreated conditions (for all γH2AX^+^ cells); ^#^ *p* < 0.05, ^##^ *p* < 0.01, ^###^ *p* < 0.001 (Kruskal-Wallis test and Dunn’s post-hoc test) compared to untreated conditions (for each distinct category of γH2AX positivity, depicted with the indicated color code). Dose range in (**A**–**C**): 100 nM CHK1i, 20 µM MRE11i for #19RES or 30 µM MRE11i for #1RES, 7.5 µM RAD51i; a.u., arbitrary units. Associated supplementary figure: Supplementary Figure S1.

We then analyzed the impact of combined inhibition of CHK1 and RAD51 (by prexasertib+B02) and CHK1 and MRE11 (by prexasertib+mirin) on DNA replication and DNA damage in CRC-SCs. Through western-blot studies, we provided evidence that these two prexasertib-based regimens promoted an elevated increase in the phosphorylation of RPA32 (pRPA32) (Figure 2A), a marker of ongoing replication stress [20,26,29], as compared to prexasertib alone. As expected, replication stress was not augmented by the administration of mirin or B02 alone (Figure 2A; Figure S1). Flow cytometry and fluorescence microscopy studies confirmed the induction of extensive DNA damage upon prexasertib-based combinatorial regimens in RES-CRC-SCs. Indeed, combined inhibition of CHK1+RAD51 and of CHK1+MRE11 significantly promoted the phosphorylation of H2AX (Figure 2B,C), a post-translational modification sensing DNA lesions best known as γH2AX [30]. In line with the induction of replication stress, in RES-CRC-SCs the two prexasertib-based combinations significantly increased the percentage of S-phase cells positive to γH2AX (Figure 2B), a marker of fork breakage occurring during elevated replication stress. Moreover, fluorescence microscopy studies revealed that a significant percentage of CRC-SCs cotreated either with CHK1+RAD51 inhibitors or with CHK1+MRE11 inhibitors displayed a diffuse γH2AX nuclear staining instead of classical nuclear foci, covering either all or a portion of the nuclei (Figure 2C). Such diffuse nuclear staining confirms the presence of excessive RS, indicating the induction of replication catastrophe.

Collectively, these findings indicate that the targeting of RAD51 or MRE11 sensitizes to CHK1 inhibitors by boosting replication stress to lethal levels. 

### 2.3. The Administration of Prexasertib Together with Mirin or B02 Alters Mitotic Timing

**Figure 3:**
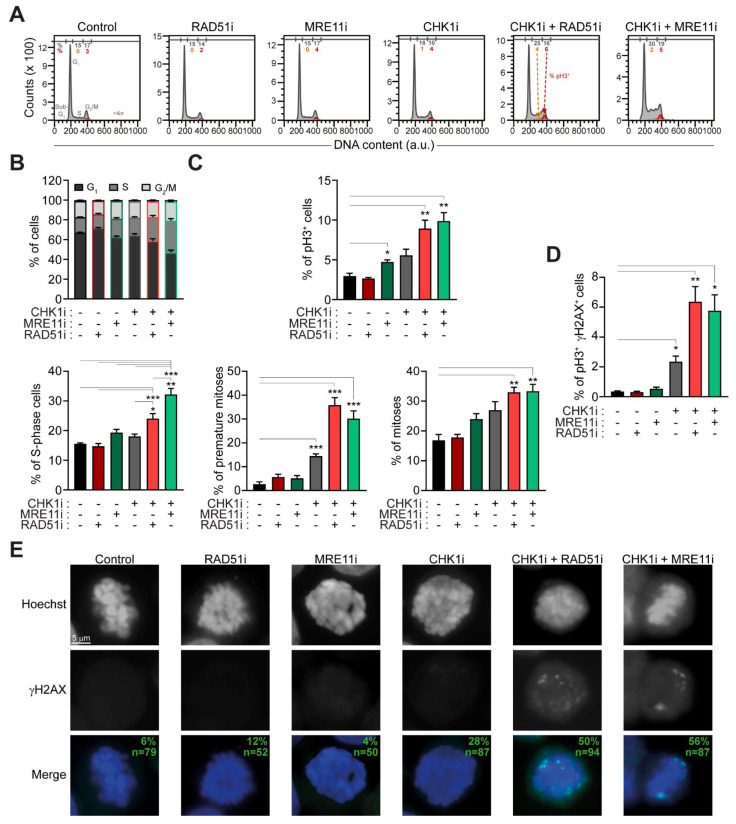
Combined inhibition of MRE11 and CHK1 or of RAD51 and CHK1 causes an accumulation of premature and damaged mitoses in CRC-SCs resistant to prexasertib. (**A**–**D**) Cytofluorimetric assessment of cell cycle profiles (**A**,**B**) or the levels of phospho(p)H3 (S10) (**A**,**C**) and/or pH3 and γH2AX (**D**) in representative RES-CRC-SC left untreated or treated for 24 h with prexasertib (CHK1i) alone or in combination with MRE11i (mirin) or RAD51i (B02), and then stained with DAPI and the appropriates antibodies. Cell cycle profiles and quantitative data (means ± SEM from six independent experiments) are reported. In (**A**), cells positive for pH3 in S-phase and G_2_/M-phase are in orange and red, respectively. Numbers indicate the percentage of corresponding events. In (**C**), the percentage of premature mitosis corresponds to the percentage of pH3^+^ cells with a DNA content lower than 4*n* among all pH3^+^ cells, while the percentage of normal mitoses corresponds to the percentage of pH3^+^ cells with a 4*n* DNA content among all cells. * *p* < 0.05, ** *p* < 0.01, *** *p* < 0.001 (one-way ANOVA and Bonferroni or Dunnett’s T3 post-hoc test in (**B**), in (**C**) for the analysis of the percentages of pH3^+^ cells and of premature mitoses, and in (**D**); Kruskal-Wallis test and Dunn’s post-hoc test in (**C**), for the analysis of the percentages of mitoses), as indicated. (**E**) Immunofluorescence detection of DNA damage in mitosis in RES-CRC-SCs left untreated or exposed for 24 h to CHK1i, alone or in combination with MRE11i or RAD51i, and then strained with DAPI and an antibody recognizing γH2AX. The panel shows representative images of (pro)metaphases (as demonstrated by the classical chromosome condensation in gray scale images) with DNA damage foci. Green numbers refer to numbers of analyzed mitoses (n) and the percentages of γH2AX^+^ mitoses pooled from five independent experiments. Dose range in (**A**–**E**): 100 nM CHK1i, 20 µM MRE11i for #19RES or 30 µM MRE11i for #1RES, 7.5 µM RAD51i; a.u., arbitrary units.

We then performed an extensive flow cytometry-mediated characterization of cell cycle progression. We provided evidence that elevated replication stress induced by MRE11+CHK1 inhibitors in RES-CRC-SCs was accompanied by changes in cell cycle profiles, manifested with a significant accumulation of cells with a DNA content between 2*n* and 4*n* (presumably S-phase cells) (Figure 3A,B). A similar accumulation (though to a lesser extent) was observed in RES-CRC-SCs treated with CHK1+RAD51 inhibitors, while prexasertib, mirin and B02 monotherapies did not significantly affect cell cycle progression (Figure 3A,B). To enter more in-depth into such an effect, we extended cell cycle analysis focusing on phosphorylated histone 3 (pH3), a marker of mitosis. By cytofluorimetry, we observed that, when combined with prexasertib, mirin and B02 significantly increased the mitotic (pH3^+^) fraction (Figure 3A,C). Intriguingly, upon exposure to prexasertib+B02 or prexasertib+mirin around 30% of cell positive for pH3^+^ did not present the classic 4*n* DNA content, but bore a DNA content between 2*n* and 4*n* (Figure 3A,C). The presence of such a high number of pH3^+^ cells with a DNA content lower than 4*n* indicates premature entry into mitosis upon cotreatment. This evidence demonstrates that combined inhibitions of CHK1 and RAD51 and of CHK1 and MRE11 in RES-CRC-SCs not only affect DNA replication but also deregulate cell cycle proliferation and mitotic timing. In particular, it suggests that prexasertib-based combinations push RES-CRC-SCs with unreplicated and/or damaged DNA into aberrant mitosis rather than causing an S-phase blockade. Again, this effect was absent or less evident in cells treated with prexasertib, B02 or mirin alone (Figure 3A–C). To further confirm this result, we performed a biparametric analysis of pH3 and γH2AX, observing a significant augmentation of the percentage of cells with double positivity for pH3 and γH2AX in RES-CRC-SCs subjected to prexasertib-based cotreatments (Figure 3D). Finally, by fluorescence microscopy studies we observed that, upon prexasertib+B02 or prexasertib+mirin cotreatment, almost half of the analyzed (pro)metaphases showed γH2AX foci (Figure 3E). This evidence confirms the presence of DNA damage in mitosis. 

Altogether, these findings indicate that the induction of replication stress by combined inhibition of CHK1 and RAD51 or of CHK1 and MRE11 culminates in premature mitosis entry and aberrant mitotic execution, thus supporting the occurrence of mitotic catastrophe [31].

### 2.4. Mitotic Catastrophe by CHK1-Based Regimens Is Executed via Caspase-Dependent Apoptosis 

**Figure 4:**
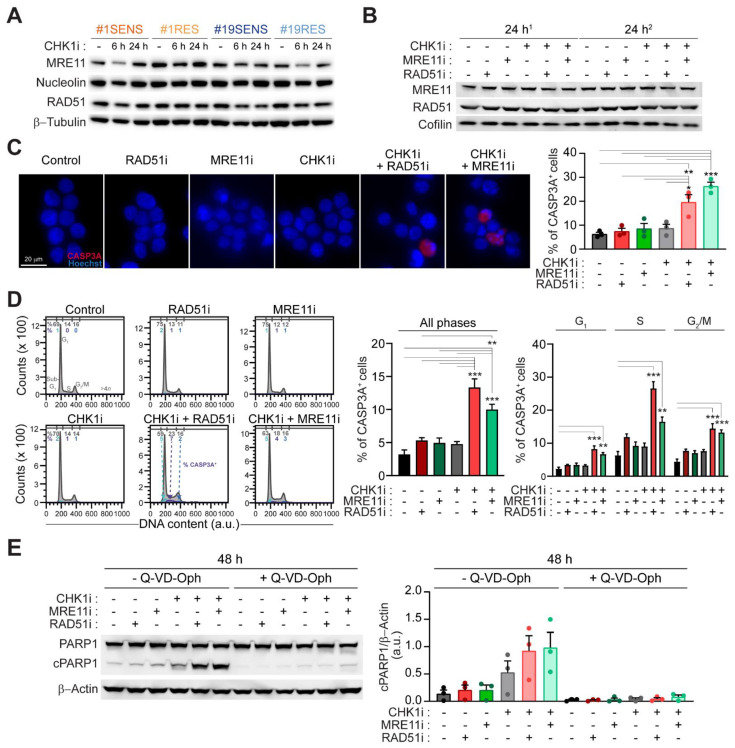
The inhibition of MRE11 or RAD51 sensitizes CRC-SCs to prexasertib by inducing a caspase-dependent mitotic catastrophe. (**A**,**B**) Western-blot analysis of MRE11 and RAD51 levels in SENS-CRC-SCs (**A**) and RES-CRC-SCs (**A**,**B**) left untreated or administrated with prexasertib (CHK1i), alone (**A**,**B**) or in combination with MRE11i (mirin) or RAD51i (B02) (**B**) (as indicated), and then stained with the appropriates antibodies. In (**B**), two independent experiments for #1RES (^1^ and ^2^) are shown. Cofilin, nucleolin and β-tubulin were used to ensure equal lane loading. Representative western-blots are reported. Quantifications are shown in Supplementary Figures S2 and S3. (**C**,**D**) Immunofluorescence- and flow cytometry-mediated detection of the activation of caspase 3 (CASP3A) in RES-CRC-SCs upon treatment for 24 h (**C**) or 48 h (**D**) with CHK1i, MRE11i, and/or RAD51i as indicated, followed by co-staining with the DNA intercalant Hoechst (**C**) or DAPI (**D**) and an anti-CASP3a antibody. In (**C**), representative images and data quantification are shown. In (**D**), cell cycle profiles (left) and quantification of the percentage of CASP3A^+^ cells among all cells (center) and the relative percentage of CASP3A^+^ cells in G_1_-, S-, G_2_/M-phase (right) are illustrated. In cell cycle profiles, positivity for CASP3A in G_1_-, S-, G_2_/M-phase is depicted in pale blue, violet and dark blue, respectively. Numbers indicate the percentages of corresponding events. In the histograms, results are expressed as means ± SEM from three or seven independent experiments in (**C**,**D**), respectively. * *p* < 0.05, ** *p* < 0.01, *** *p* < 0.001 (one-way ANOVA and Bonferroni post-hoc test) compared to untreated conditions. (**E**) Western-blot analysis in RES-CRC-SCs treated for 48 h as indicated, using an antibody directed against PARP1, also recognizing the cleaved (c) form. β-Actin was used to monitor equal lane loading. Representative western-blots are shown (see also Supplementary Figure S4). Quantification of data, expressed as means ± SEM, and individual data points are from three independent experiments. cPARP1, cleaved PARP1. Dose range in **A**–**E**: 100 nM CHK1i, 20 µM MRE11i for #19RES or 30 µM MRE11i for #1RES, 15 µM Q-VD-Oph, 7.5 µM RAD51i; a.u., arbitrary units. Associated supplementary figures: Supplementary Figures S2–S4.

We then explored the mechanism of cell killing by prexasertib+mirin and prexasertib+B02. Through western-blot, we observed similar constitutive levels of MRE11 and RAD51 in both SENS- and RES-CRC-SCs (Figure 4A; Figure S2). Moreover, no significant modulation of the level of these proteins was detected upon administration of CHK1 inhibitor, alone or in combination with MRE11 and/or RAD51 inhibitors (Figure 4B; Figure S3), ruling out a mechanism of sensitization mediated by the upregulation of MRE11 and/or RAD51. We then investigated whether the demise of RES-CRC-SCs undergoing mitotic catastrophe occurred via apoptosis, focusing on the involvement of caspases. Immunofluorescence microscopy studies of caspase 3 (CASP3) activation revealed that both prexasertib+B02 and prexasertib+mirin induced a significant increase in the level of cleaved CASP3 (Figure 4C), the activated form of this protease during apoptosis. On the contrary, monotherapies with these drugs did not significantly activate CASP3 (Figure 4C). Similar findings were obtained through flow cytometry analyses. In these studies, we observed a global increase of CASP3 activation in CRC-SCs subjected to prexasertib-based regimens, and in particular of CASP3A^+^ cells displaying a DNA content between 2*n* and 4*n* (which include premature mitoses) and a 4*n* DNA content (which include apparent normal mitoses) (Figure 4D). In line with CASP3 involvement, combined inhibitions of CHK1 with RAD51 or CHK1 with MRE11 induced the cleavage of PARP1 (Figure 4E; Figure S4), a downstream target of activated CASP3. Consistently, PARP1 cleavage was completely abolished in CRC-SCs subjected to these prexasertib-based combinations by the administration of the caspase inhibitor Q-VD-Oph (Figure 4E; Figure S4). 

Collectively, these results indicate that combined inhibition of CHK1 and MRE11 or RAD51 ultimately kills CRC-SCs via caspase-dependent apoptosis.

### 2.5. Prexasertib in Combination with RAD51 or MRE11 Inhibitors Disrupts 3D Tumorsphere Organization and Growth

**Figure 5:**
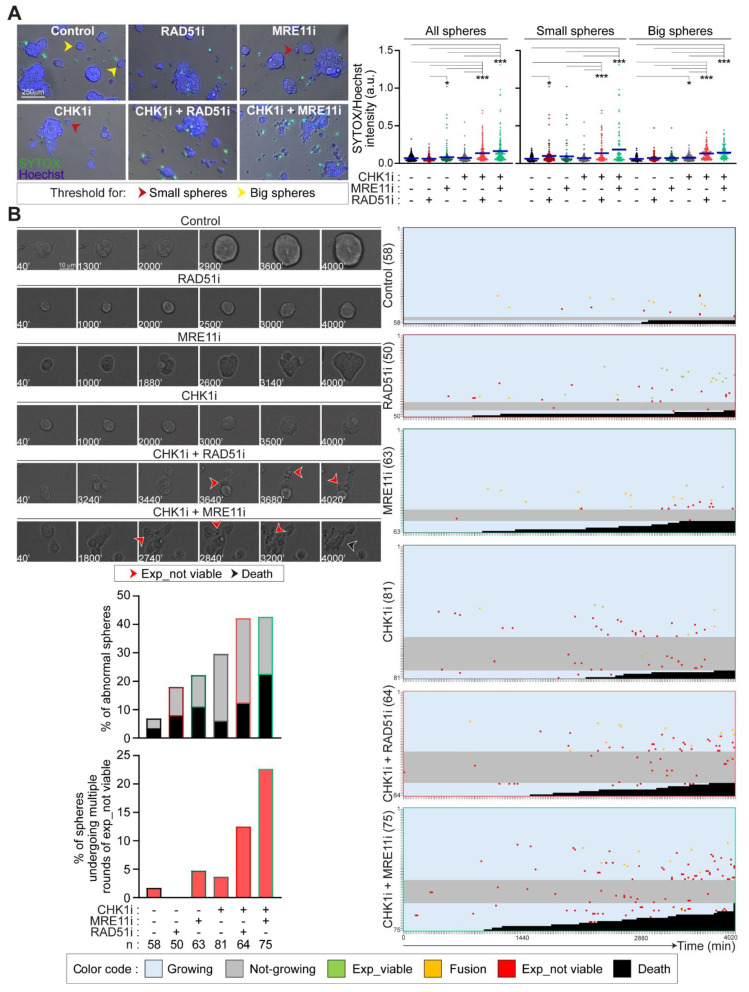
The cooperation between MRE11+CHK1 and RAD51+CHK1 is essential for the survival of CRC-SCs resistant to prexasertib. (**A**) Live cell microscopy assessment of apoptosis induction in representative RES-CRC-SCs left untreated or exposed for 48 h to prexasertib (CHK1i), MRE11i (mirin) and/or RAD51i (B02) as indicated, and then incubated for 10 min with SYTOX (which incorporates only dead cells) and the DNA dye Hoechst. SYTOX incorporation (as a parameter of regulated cell death activation) was evaluated by live fluorescence microscopy and image analysis. Nuclear fluorescence was used to discriminate spheres (area higher than 1000 pixels) from single cells or small cell aggregates (area lower than 1000 pixels) on the basis of the threshold indicated with a red arrow point (1000 pixels). Spheres were further classified in small spheres (area comprised between 1000 and 3000 pixels) and big spheres (area > 3000 pixels) on the basis of the threshold indicated with a yellow arrow point (approximately 3000 pixels). Green intensity means (i.e., SYTOX incorporation) were quantified in such spheres. Data, expressed as ratios of SYTOX/Hoechst intensity, are a pool of two independent experiments performed on distinct RES-CRC-SCs, and are shown as box-plots with means and individual data points. In the box-plot on the right, spheres are divided in small and big spheres. * *p* < 0.05, *** *p* < 0.001 (Kruskal-Wallis ANOVA and Dunn’s post-hoc test). (**B**) Live cell videomicroscopy analysis of one representative RES-CRC-SCs (#19RES) grown as 3D tumorspheres, left untreated or exposed to CHK1i, either alone or in combination with MRE11i or RAD51i. Images were taken every 20 min for up to 67 h (see Materials and Methods). Representative frames of the fate of one sphere per condition are shown, with numbers referring to the time passed from the beginning of the recording. Expulsion of one or more dead/inert cells or of one or more dead/inert cell aggregates are depicted with a red arrow point (**Exp_not viable**), while sphere disaggregation and/or death with a dark arrow point (**Death**). The fate of all spheres analyzed (at least 50 spheres per condition) are represented on the right using the indicated color code. In such “sphere fate profile”, each single sphere is depicted by a hyphen, with the first and last spheres analyzed also illustrated by a number. The following events are included: (i) expulsion of one or more viable cells or cell aggregates (**Exp_viable**; in green), (ii) sphere fusion (**Fusion**; in orange), (iii) expulsion of one or more cells or cell aggregates with an apoptotic or inert morphology (**Exp_not viable**; in red), (iv) sphere disaggregation and/or death (**Death**; in dark). The growth of each sphere was determined by measuring the area of the sphere at the beginning and at the end of the recording, and then calculating the ratio of the latter on the former. We considered as growing spheres only those with a growth ratio higher than 1.3. **Growing** and **not-growing** spheres are respectively colored in blue and grey. Numbers indicate the total number of spheres for each condition, counted in two separate videos. In the histogram in the panel, results are expressed as percentages of abnormal spheres (comprising spheres not growing and/or disaggregated/dead spheres) on the top and of spheres undergoing multiple (i.e., more than three) rounds of expulsion of inert/non-viable cells or cell aggregates on the bottom. For more information about the experiments, categories, and exclusion criteria see Materials and Methods. See also Supplementary Videos. Dose range in A–D: 100 nM CHK1i, 20 µM MRE11i for #19RES or 30 µM MRE11i for #1RES, 7.5 µM RAD51i; a.u., arbitrary units. Associated supplementary figure: Supplementary Figure S5.

We then analyzed the impact of the coinhibition of CHK1 and RAD51 or MRE11 on the organization and survival of CRC-SCs grown in vitro as 3D tumorspheres. We first performed live fluorescence microscopy in RES-CRC-SCs left untreated or administered with CHK1, RAD51 and/or MRE11 inhibitors, either alone or in combination. After treatment, RES-CRC-SCs were co-incubated with SYTOX, a vital dye incorporated by cells undergoing regulated cell death due to loss of membrane integrity, together with Hoechst 33342 to visualize the nuclei (Figure 5A). Image analysis of SYTOX incorporation confirmed that combined treatment of prexasertib with either B02 or mirin induced a high level of apoptosis in tumorspheres as compared to prexasertib, B02 or mirin monotherapies (Figure 5A). This evidence demonstrates the occurrence of apoptosis in CRC-SC tumorspheres. To explore more in-depth this phenomenon, we performed live videomicroscopy studies monitoring RES-CRC-SCs grown as spheres for approximately 67 h upon drug administration. In this analysis, we provided evidence that the two prexasertib-based combinations affected 3D tumorsphere growth and organization. Indeed, prexasertib+mirin (and to a lesser extent) prexasertib+B02 induced disaggregation of a significant number of spheres, manifested with sphere demise or dissolution (Figure 5B). Moreover, both combinations (and in particular prexasertib+B02) decelerated the growth of viable spheres (i.e., those that did not undergo disaggregation) as shown by the mild increase in sphere diameter at the end of the experimentation in these conditions, a phenomenon also observed with prexasertib (Figure 5B). Further confirming the effect on sphere organization, combined administration of prexasertib with B02 or mirin (but not or less monotherapies) promoted the occurrence of multiple rounds of expulsion of cells/spheres displaying an apoptotic morphology (Figure 5B).

Collectively, these results demonstrate that mirin and B02 sensitize CRC-SCs to prexasertib by disrupting tumorsphere organization and growth.

## 3. Discussion

The ATR-CHK1 axis is particularly relevant for the survival of CSCs, which often display high levels of replication stress, and, accordingly, ATR and CHK1 inhibitors are being explored for the design of anti-CSC therapies [20,22,23]. Moreover, the antineoplastic activity of ATR and CHK1 inhibitors, including prexasertib, is currently investigated in ongoing clinical trials (https://www.clinicaltrials.gov/). However, a significant fraction of CSCs is intrinsically resistant to these therapies, and resistance mechanisms also emerge. Here, we provided evidence that the targeting of either MRE11 or RAD51 sensitizes primary CRC-SCs to the CHK1 inhibitor prexasertib by inducing a mitotic catastrophe process culminating in caspase-dependent cell death.

Results from this study indicate that sensitization to prexasertib by RAD51 or MRE11 inhibitors involves the boost of replication stress, which reinstates CRC-SC dependence on the function of the ATR-CHK1 pathway of the replication stress response. This is in line with previous evidence showing enhanced anti-CSC activity of ATR-CHK1 inhibitors due to the induction of replication stress by agents including (but not limited to) gemcitabine, irinotecan and inhibitors of PARP1, RRM2 or WEE1 [20,25,26,32,33,34]. However, here, we showed that the two identified prexasertib-based regimens exerted a rather broad impact on the DDR of CRC-SCs, simultaneously impairing several cellular processes for the preservation of genomic stability. Indeed, concomitant inhibition of CHK1 and RAD51 or MRE11 deregulated not only the DNA replication process but also cell cycle progression and ultimately cell division, resulting in a general reorientation of the DDR toward cytotoxicity. This evidence is relevant for cancer therapy, as it suggests that prexasertib-based combinations can potentially overcome the reported heterogeneity in the therapeutic response of CSCs, which constitutes one major challenge in cancer therapy. In particular, CSC sensitivity to ATR-CHK1 inhibitors could be limited by a variety of cytoprotective mechanisms characterizing CSCs, encompassing (among others) strong DNA damage signaling and repair, cell cycle checkpoint proficiency, elevated tolerance to DNA damage, and limited apoptosis induction (reviewed in [10]). As a consequence, therapeutic regimens that simultaneously target multiple of these aspects—like those based on the combination of CHK1+MRE11 or CHK1+RAD51 inhibitors—can be particularly effective in eradicating CSCs, also limiting the potential development of acquired resistance. 

At the mechanistic level, in this study, we demonstrated that combined inhibition of CHK1 and either MRE11 or RAD51 leads to uncontrolled cell cycle progression and untimely mitotic entry of cells with ongoing replication stress. Usually, the proliferation of replication stressed cells is limited by the activation of the intra-S and G_2_/M checkpoint, both of which depend on the ATR-CHK1 axis [35,36] and, so, are silenced on its abrogation. Consistently, under prexasertib-based regimens, we found illicit proliferation and mitotic entry of CRC-SCs with unreplicated and damaged DNA, as shown by the presence of premature mitoses (pH3^+^ cells with DNA content between 2*n* and 4*n*) and of mitotic damage (mitotic cells displaying γH2AX foci). Hence, we propose a model in which pre-mitotic defects linked to replication stress emerging upon CHK1+MRE11 or CHK1+RAD51 inhibition are eventually transmitted into mitosis, threatening genomic stability, affecting sister chromatid segregation, and resulting in the activation of mitotic catastrophe. 

At this regard, the inhibition of RAD51 or MRE11 may constitute the initiating factor of prexasertib sensitization. Indeed, RAD51 is a homologous recombination (HR) player also contributing to the stabilization, regression and restart of stressed DNA replication forks [37,38,39,40,41], meaning that its inhibition can impair the replication stress response. Similar considerations apply to MRE11, which has a pleiotropic impact on the DDR. In particular, a controlled and limited resection by MRE11 is crucial for the efficient restart or repriming of stalled forks [42,43]. In this context, uncontrolled fork degradation by MRE11, which occurs in conditions of fork instability or unprotection [44,45], is restricted by factors including RAD51 [46]. Moreover, MRE11 as a component of the MRE11-RAD50-NBS1 (MRN) complex has roles in the G_2_/M checkpoint, regulating the interphase-prophase transition, and also acts in sister chromatid segregation during mitosis [47,48,49]. Further studies will uncover the precise mechanisms through which RAD51 or MRE11 inhibitors affect mitosis entry and execution upon concurrent CHK1 inhibition. Irrespective of these mechanistic unknowns, we demonstrated that mitotic catastrophe by RAD51+CHK1 or MRE11+CHK1 inhibitors resulted in tumorsphere disaggregation and CRC-SC death via caspase-dependent apoptosis. The particular proneness of CRC-SCs to mitotic catastrophe suggested by this and our previous study [26] can be potentially exploited for the design of effective colorectal cancer therapy.

## 4. Materials and Methods

### 4.1. Cell Lines, Culture Conditions and Chemicals

Media, supplements and plasticware for cell culture were supplied by Thermo Fisher Scientific (Waltham, MA, USA), Sigma-Aldrich (Millipore-Sigma, Merck Group, St. Louis, MO, USA) and Corning Life Sciences (Corning, NY, USA). Colorectal cancer cells (CRC-SCs) used in this study were previously isolated and established from patient sample following the standards of the institutional ethics committee on human experimentation (authorization no. CE5ISS09/282) as reported in [50]. Informed consent was requested in this previous study. The methods for CSC authentication, validation and cultivation are reported in [20,50], while generation of CRC-SCs resistant to CHK1 inhibitors (RES-CRC-SCs) is described in [26]. Adavosertib (#S1525), B02 (#S8434), KU-60019 (#S1570), mirin (#S8096), NU7026 (#S2893), prexasertib (#S7178), Q-VD-Oph (#S7311), triapine (#S7470), and VE-821 (#S8007) were supplied by Selleck Chemicals (Houston, TX, USA), while dimethyl sulfoxide (DMSO, #5879) by Sigma-Aldrich. Twenty-four hours before each experimentation, CRC-SCs were dissociated at single cells, then counted and seeded in specific multi-well plates. For live microscopy and videomicroscopy studies, cell seeding was carried out 48 h before the treatment to allow tumorsphere formation.

### 4.2. Cell Proliferation and Viability

To assess proliferation and survival, CRC-SCs were dissociated at single cells and seeded at a density of 6–8 × 10^3^ cells per well in 96-well plates, in a volume of 100 µL of medium per well. After 24 h, CRC-SCs were treated according to specific experimentations and cell viability/proliferation was determined by assessing ATP levels via CellTiter-Glo^®^ Luminescent Cell Viability Assay (#G7572, Promega, Madison, WI, USA) with a multimode microplate reader (DTX-880; Beckman Coulter, Brea, CA, USA). Experiments were carried out in triplicate and repeated at least three independent times, with results expressed as means of triplicate values or of duplicate values when we encountered technical problems with one replicate, as reported in the statistical procedures. The heatmap in Figure 1A illustrates the percentage of viable RES-CRC-SCs determined via CellTiter-Glo^®^ Assay upon normalization of treated on untreated conditions using data reported in Supplementary Table S1. Drug sensitivity heatmap was generated using a Python script (Python Software Foundation, Wilmington, DE, USA; https://www.python.org/) and the Seaborn library (10.5281/zenodo.592845). The heatmap scale goes from 100 (blue; i.e., RES-CRC-SCs are all alive) to 25 (dark red) in consideration of the fact that 25% is the minimum of viability obtained for RES-CRC-SCs using the combination prexasertib+triapine.

### 4.3. Cytofluorometric Studies

Flow cytometry studies aimed at determining cell cycle profile, mitosis fraction, DNA damage and/or apoptosis of CRC-SCs were performed as previously reported [26]. The following primary antibodies were used: cleaved caspase 3 (CASP3; #9661) and phospho-histone H3 (pH3; #3377) from Cell Signaling Technology (Danvers, MA, USA), and γH2AX (#05-636) from Merck Millipore. Secondary antibodies used were: Alexa Fluor^®^ 488-goat anti-mouse and 647-donkey anti-rabbit ((#A-21121 and #A-31573). These antibodies were provided by Thermo Scientific. Antibody dilutions and the Research Resource Identifiers (RRIDs) are in [26]. The DNA intercalant DAPI (#D1306, from Thermo Scientific) at 10 µM was employed to stain the DNA. The samples were acquired by a BD FACSCelesta^TM^ flow cytometer (BD Biosciences, BD, Franklin Lakes, NJ). Data and statistical analyses were performed with the FlowJo software (FlowJo LLC, BD), gating on events showing normal forward and side scatter (FSC and SSC) parameters, on singlets and (for cell cycle analysis) upon exclusion of the sub-G_1_ fraction. 

### 4.4. Immunofluorescence Studies

Immunofluorescence studies aimed at detecting DNA damage markers in CRC-SCs were performed as reported in [26]. Briefly, upon permeabilization, the cells were blocked for 30 min in 5% (*w*/*v*) BSA, 5% FBS and 5% normal goat serum (NGS) in PBS. Thereafter, samples were incubated with primary antibodies directed against cleaved CASP3 (1:400; #9661) or γH2AX (S139) (1:250; #05-636) at 4 °C. After overnight incubation, slides were stained with secondary Alexa Fluor conjugates (1:500) together with 10 µM Hoechst 33342 (#H1399). Fluorescence images were visualized, analyzed and captured with a Leica DMI3000 B microscope, using a 40× objective (HCX PL Fluotar, AN 0.60) or a 100× objective (HCX PL Fluotar, AN 1.3), the Leica DFC 310FX camera, and the LAS X software (all from Leica Microsystems, Wetzlar, Germany). Analyses were carried out directly at the microscope or using the ImageJ v1.8 software (National Institute of Health, Bethesda, MD, USA; https://imagej.nih.gov/ij/). To determine γH2AX positivity, at least 61 cells per condition and per experiment were analyzed for a total of (at least) 743 cells (in five experiments). We considered three categories of positivity: (1) “focal” staining, when the cell presents > five γH2AX foci, (2) “partially-diffuse” staining, when the cell presents a diffuse γH2AX positivity covering between less than half of the nuclei, and (3) “pan-nuclear” staining, when the cell presents a diffuse γH2AX positivity covering more than half of the nuclei (see Figure 2). In Figure 3, cells were recognized as mitoses on morphological basis upon nuclear staining with Hoechst 33342. For CASP3 activation analysis, at least 261 cells per condition and per experiment were analyzed for a total of (at least) 923 cells (for three experiments). In such automated analysis, cells were considered positive for CASP3 when signal intensity calculated with ImageJ was above a predefined threshold size to account for background noise.

### 4.5. Immunoblotting

To detect protein levels, CRC-SCs were recollected, lysed, and subjected to western-blot as reported in [20]. Membranes were incubated with primary antibodies directed against MRE11 (1:1000; #sc-22767; RRID:AB_2145247 and #sc-135992; RRID:AB_2145244) from Santa Cruz Biotechnology (Dallas, TX, USA), PARP1 (#9542) from Cell Signaling Technology, RAD51 (1:1000; #sc-398587; RRID: AB_2756353 and #sc-377467) from Santa Cruz Biotechnology, RPA32 (#A303-874A) from Bethyl Laboratories (Montgomery, TX, USA) and pRPA32 (S4/S8) (#A300-245A) from Bethyl Laboratories, as well as with antibodies recognizing β-actin (1:2000; #A5441; RRID:AB_476744), cofilin (#3318, Cell Signaling Technology), nucleolin (1:1000; #14574; Cell Signaling Technology; RRID:AB_2798519), or β-tubulin (#T4026). After overnight incubation with primary antibodies and washes, membranes were incubated for 1 h at RT with the appropriate horseradish secondary antibodies conjugated to peroxidase: the donkey anti-rabbit IgG (#GENA934 or RRID:AB_772206), mouse anti-goat IgG (#sc-2354, Santa Cruz; RRID:AB_628490), or sheep anti-mouse IgG (#GENA931). When not specified, antibody dilutions and RRIDs can be found in [26]. Chemiluminescence imaging was carried out with the Fujifilm LAS-4000 luminescent image analyzer (GE Healthcare, Chicago, IL, USA), or the G:Box Chemi-XX9 using the GeneSys Software v1.5.6 (Synoptics, Cambridge, UK). At least two independent experiments were carried out. Representative Western-blots are shown in the figure together with the densitometry plus quantification, which were performed with ImageJ v1.8 software. Full blots are presented in Supplementary Figures S1–S4.

### 4.6. SYTOX Incorporation

To determine the induction of apoptosis, CRC-SCs dissociated at single cells were resuspended in 90% CSC growth medium + 10% Matrigel Basement Membrane Matrix (Matrigel, #354230, Corning), at a density of 3.5 × 10^5^ cells in 500 µL. CRC-SCs were then seeded in 24-well plates, incubated for 24 h in standard culture condition and finally treated for 48 h with prexasertib, mirin and/or B02, alone or in combination, as reported in figure legends. After the (co)treatment, cells were incubated with the vital dye SYOX^TM^ (at 5 µM; #S7020, Thermo Scientific) together with the DNA dye Hoechst 33342 (at 8 µM) for 10 min. Cells were immediately analyzed using the Leica DMI3000 B fluorescence microscope equipped with a 20× objective (HCX PL Fluotar AN 0.40) (see above for further details). A number of at least eight randomly selected images were analyzed with ImageJ v1.8 software (Fiji version) as follow. Briefly, nuclear fluorescence was used to discriminate single cells or small cell aggregate from spheres (threshold area: 1000 pixels), on which the green intensity (corresponding to dead cells) means were quantified and finally divided on Hoechst intensity. A minimum of 190 spheres per condition were analyzed. 

### 4.7. Videomicroscopy

To determine the impact of prexasertib-based combinations on 3D tumorspheres, CRC-SCs were dissociated at single cells and then resuspended in 80% CSC medium + 20% Matrigel, at a density of 1 × 10^5^ cells in a drop of 50 µL, and seeded in 24-well plates in a final volume of 500 µL. Upon 48 h incubation in standard culture conditions, cells were subjected to live videomicroscopy analysis using a Nikon LIPSI system (Nikon, Minato-ku, Tokyo, Japan) equipped with IRIS 15 photometrics camera allowing for standard culture cultivation. Images were taken every 20 min for up to 67 h, with a 20× long-range objective (S-PLAN AN 0.4) and analyzed with ImageJ v1.8 software (Fiji version). The following criteria of exclusion were adopted. Spheres were excluded from analysis when: (1) the distance from another sphere was less than 13 µm, so to avoid problems due to excessive rounds of sphere fusion; (2) underwent more than one round of fusion with other spheres, thus becoming too big to be analyzed; (3) had a length for one of the two orthogonal diameters (which have been arbitrarily chosen in the first frame of the movie) less than 13 µm; (4) had a length for the first orthogonal diameter <30 µm and the second orthogonal diameter < 40µm, (5) the fate was not clear to define; and (6) their demise occurred within the first 12 h of the video recording (and thus presumably not caused by the treatment). The following events were retained as important: (1) expulsion of apparently viable cell(s) or cell aggregate(s), as determined by morphological criteria and the capability to divide and/or grow, (2) expulsion of apparently non-viable or inert cell(s) or cell aggregate(s), as determined by morphological criteria, (3) fusion with another viable sphere, and (4) disaggregation and death of the spheres, as determined by morphological criteria. In the histograms of Figure 5B, results are expressed as percentages of abnormal spheres (comprising bot disaggregated spheres and not-growing spheres) and of spheres undergoing multiple rounds of expulsion of non-viable cells/cell aggregates. In this figure, the growth of each sphere was determined by measuring the sphere area at the beginning and at the end of the recording, and then dividing the latter on the former value. Spheres were considered as expanding when the ratio was higher than 1.3.

### 4.8. Statistical Procedures

All the experiments were repeated in at least three independent instances, but in case of absent sub-significant trends, the sample size was increased to more than three (see figure legends or dedicated material method sections for the exact sample size, the presence of replicates, and data/replicate exclusion criteria). We evaluated the variance equality using the F-test or the Brown-Forsythe test, when appropriate. We calculated the Shapiro-Wilk normality test for all the continuous variables. For the variables that are normally distributed and according to the number of groups compared, we used unpaired Student T test or Welch’s unpaired T-test, and one-way ANOVA followed by Bonferroni post-hoc test or Brown-Forsythe and Welch one-way ANOVA followed by Dunnett’s T3 post-hoc test, when appropriate. For the variables that are not normally distributed or for less than three independent experiments, we applied Mann-Whitney test or Kruskal-Wallis test followed by Dunn’s post-hoc test, according to the number of groups. Data (including normalization) were calculated and visualized as reported in [26]. The following softwares were used: Microsoft Excel (Microsoft, Redmond, WA, USA), Prism (v8.3.0, GraphPad Software, San Diego, CA, USA) and SPSS (SPSS v.21, SPSS Inc-IBM, Chicago, IL, USA).

## 5. Conclusions

In this study we identified RAD51 and MRE11 as two targets whose inhibition increases the sensitivity of CRC-SCs to CHK1 inhibitors. We also characterized the mechanism of CSC killing by CHK1+RAD51 and CHK1+MRE11 inhibitors, showing that it involves the induction of replication stress followed by progression of replication stressed CRC-SCs through the interphase and their premature entry into mitosis, ultimately leading to caspase-dependent mitotic catastrophe. These results support the future clinical development of prexasertib-mediated regimens in colorectal cancer patients.

## Data Availability

The materials and data are available from the corresponding authors.

## Supplementary Materials

The following are available online at https://www.mdpi.com/article/10.3390/cancers13081957/s1. Supplementary Figure S1. Analysis of replication stress in RES-CRC-SCs exposed to prexasertib-based regimens. Related to Figure 2. Supplementary Figure S2. Analysis of MRE11 or RAD51 levels in SENS-CRC-SCs and RES-CRC-SCs. Related to Figure 4. Supplementary Figure S3. Analysis of MRE11 or RAD51 levels in SENS-CRC-SCs and RES-CRC-SCs. Related to Figure 4. Supplementary Figure S4. Analysis of PARP1 cleavage in RES-CRC-SCs. Related to Figure 4. Supplementary Figure S5. CHK1 cooperates with MRE11 or RAD51 for RES-CRC-SC survival. Related to Figure 5. Supplementary Movies 1–12. Impact of MRE11+CHK1 inhibition and RAD51+CHK1 inhibition on CRC-SC organization, proliferation and survival. Related to Figure 5. Supplementary Table S1. Raw data of the heatmap presented in Figure 1A. Supplementary Information.
