# Supplementary material for: The Targeting of MRE11 or RAD51 Sensitizes Colorectal Cancer Stem Cells to CHK1 Inhibition
Source: Cancers (Basel). 2021 Apr 19;13(8):1957. doi: 10.3390/cancers13081957 (PMC8073980; doi:10.3390/cancers13081957)
Supplement: Supplementary file 1 [file cancers-13-01957-s001.zip › Supplementary information Mattiello et al.pdf]

## Supplementary Figure legends

**Supplementary Figure S1. Analysis of replication stress in RES-CRC-SCs exposed to prexasertib-based regimens. Related to Figure 2.**

The figure reports the full western-blot presented in **Figure 2A**. The western-blot membrane was cut, and the lower part was immunostained with pRPA32 and RPA32 antibodies (framed in green) while the higher part with a nucleolin antibody (framed in blue).

**Supplementary Figure S2. Analysis of MRE11 or RAD51 levels in SENS-CRC-SCs and RES-CRC-SCs. Related to Figure 4.**

The figure reports the full images (**A**,**B**) and the quantification (**C**, **D**) of the western-blots presented in **Figure 4A**. In **A**, the western-blot membrane was cut and immunostained with RAD51 and β-tubulin antibodies (framed in green). In **B**, the western-blot membrane was immunostained with MRE11 and nucleolin antibodies (framed in green).

**Supplementary Figure S3. Analysis of MRE11 or RAD51 levels in SENS-CRC-SCs and RES-CRC-SCs. Related to Figure 4.**

The figure reports the full images (**A**) and the quantification (**B**) of the western-blots presented in **Figure 4B**. In **A**, the western-blot membrane was cut, and the lower part was immunostained with cofilin and RAD51 antibodies (framed in green), while the higher part with a MRE11 antibody (framed in blue).

**Supplementary Figure S4. Analysis of PARP1 cleavage in RES-CRC-SCs. Related to Figure 4.**

The figure reports the full images of the western-blots presented in **Figure 4E**. The western-blot membrane was cut, and the lower part was immunostained with a β-tubulin antibody (framed in

green), while the higher part with a PARP1 antibody (framed in blue) also recognizing cleaved PARP1 (cPARP1)

**Supplementary Figure S5. CHK1 cooperates with MRE11 or RAD51 for RES-CRC-SC survival. Related to Figure 5.**

(**A**) Live assessment of apoptosis induction in RES-CRC-SCs left untreated or exposed for 48h to CHK1i, MRE11i and/or RAD51i as indicated, and then incubated with SYTOX (which incorporates only in dead cells) and the DNA dye Hoechst for 10min. Sphere identification and analysis were performed as reported in **Figure 5A** and **Materials and Methods**.

(**B**) Live videomicroscopy analysis of RES-CRC-SCs (#19RES) grown as 3d tumorspheres left untreated or exposed to CHK1i, either alone or in combination with MRE11i or RAD51i. Images were taken every 20 min for up to 67h. Representative frames of one sphere per condition are shown, with numbers referring to the time passed from the beginning of the recording. For more detail, s*ee* **Figure 5B** and **Materials and Methods.** *See* also **Supplementary videos 7-12.**

**Supplementary Movies 1-12. Impact of MRE11+CHK1 inhibition and RAD51+CHK1 inhibition on CRC-SC organization, proliferation and survival. Related to Figure 5.**

Full movies representing the fate of representative CRC-SC spheres upon treatment with CHK1i alone in combination with MRE11i or RAD51i, from which the frames reported in **Figure 5 (Supplementary Movies 1-6)** and **Supplementary Figure S5 (Supplementary Movies 6-12)** are taken.

**Supplementary Movie 1**: untreated CRC-SC sphere growing normally

**Supplementary Movie 2**: CRC-SC sphere treated with RAD51i growing normally

**Supplementary Movie 3**: CRC-SC sphere treated with MRE11i growing normally

**Supplementary Movie 4**: CRC-SC sphere treated with prexasertib growing normally

**Supplementary Movie 5**: CRC-SC sphere treated with prexasertib+RAD51i expelling unviable cells or cell aggregates

**Supplementary Movie 6**: CRC-SC sphere (the sphere on the top) treated with prexasertib+MRE11i expelling unviable cells or cell aggregates and then undergoing disaggregation

**Supplementary Movie 7**: untreated CRC-SC sphere growing normally

**Supplementary Movie 8**: CRC-SC sphere treated with RAD51i expelling viable cells or cell aggregates

**Supplementary Movie 9**: CRC-SC spheres treated with MRE11i fusing with each other

**Supplementary Movie 10**: CRC-SC sphere treated with prexasertib expelling unviable cells/cell aggregates

**Supplementary Movie 11**: CRC-SC sphere treated with prexasertib+RAD51i expelling unviable cells/cell aggregates, and then undergoing disaggregation

**Supplementary Movie 12**: CRC-SC sphere (the central one) treated with prexasertib+MRE11i expelling unviable cells or cell aggregates, and then undergoing fusion with another sphere and finally disaggregation

*See* the legends of **Figure 5** and **Supplementary Figure S5** and **Materials and Methods** for further details.

**Supplementary Table S1. Raw data of the heatmap presented in Figure 1A.**
