# Supplementary material for: The Targeting of MRE11 or RAD51 Sensitizes Colorectal Cancer Stem Cells to CHK1 Inhibition
Source: Cancers (Basel). 2021 Apr 19;13(8):1957. doi: 10.3390/cancers13081957 (PMC8073980; doi:10.3390/cancers13081957)
Supplement: Supplementary file 1 [file cancers-13-01957-s001.zip › Supplementary Table 1 Mattiello et al.pdf]

Supplementary Table S1. Raw data of the heatmap presented in Figure 1A

EXPERIMENT : Seinsitization to Prexasertib (100 nM)
ENDPOINT : Evaluation of % of viable cells by CellTiter Glo 72h upon treatment
NOTE : Number of independent experiments (n) =3
For each independent experiment, the reults are normalized over untreated/control condition.

			**Independent experiments**			
			**1**	**2**	**3**	**Mean**
Target	**RAD51**	Untreated (NT)	100	100	100	100
Drug	**B02**	Prexasertib	92	101	84	92
Concentration	10 µM	B02	81	72	76	76
		Prexasertib + B02	50	50	52	51
Target	**MRE11**	NT	100	100	100	100
Drug	**Mirin**	Prexasertib	93	76	82	84
Concentration	25 µM	Mirin	92	93	67	84
		Prexasertib + Mirin	74	59	42	58
Target	**Topoisomerase I**	NT	100	100	100	100
Drug	**Irinotecan**	Prexasertib	102	94	102	100
Concentration	1 µM	Irinotecan	94	84	95	91
		Prexasertib + Irinotecan	65	67	79	70
Target	**WEE1**	NT	100	100	100	100
Drug	**Adavosertib**	Prexasertib	87	79	97	88
Concentration	100 nM	Adavosertib	77	70	102	83
		Prexasertib + Adavosertib	37	25	40	34
Target	**RRM2**	NT	100	100	100	100
Drug	**Triapine**	Prexasertib	88	89	94	91
Concentration	200 nM	Triapine	101	89	116	102
		Prexasertib + Triapine	30	27	19	25
Target	**ATR**	NT	100	100	100	100
Drug	**VE-821**	Prexasertib	79	85	96	87
Concentration	15 µM	VE-821	68	75	84	76
		Prexasertib + VE-821	60	75	80	72
Target	**DNA-PK**	NT	100	100	100	100
Drug	**NU7026**	Prexasertib	85	86	87	86
Concentration	50 µM	NU7026	88	88	94	90
		Prexasertib + NU7026	81	79	86	82
Target	**ATM**	NT	100	100	100	100
Drug	**KU-60019**	Prexasertib	87	89	83	86
Concentration	5 µM	KU-60019	78	91	86	85
		Prexasertib + KU-60019	76	78	77	77
