# Supplementary figures and images for: The Targeting of MRE11 or RAD51 Sensitizes Colorectal Cancer Stem Cells to CHK1 Inhibition
Source: Cancers (Basel). 2021 Apr 19;13(8):1957. doi: 10.3390/cancers13081957 (PMC8073980; doi:10.3390/cancers13081957)

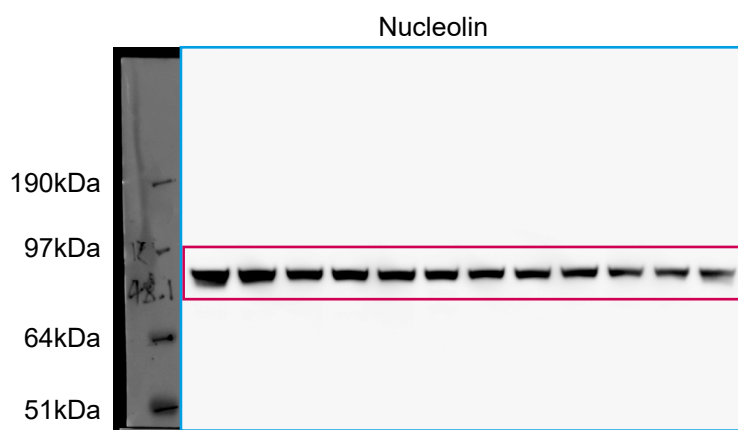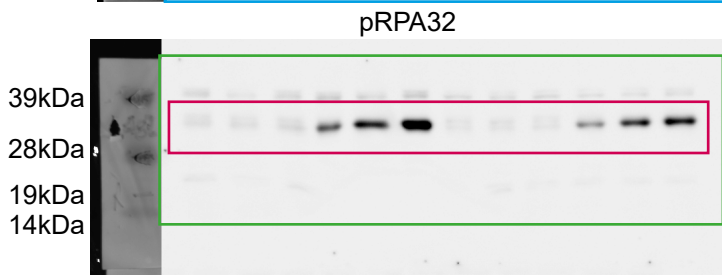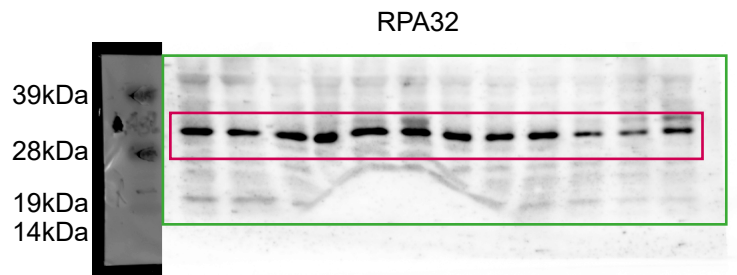

**FIGURE S1**

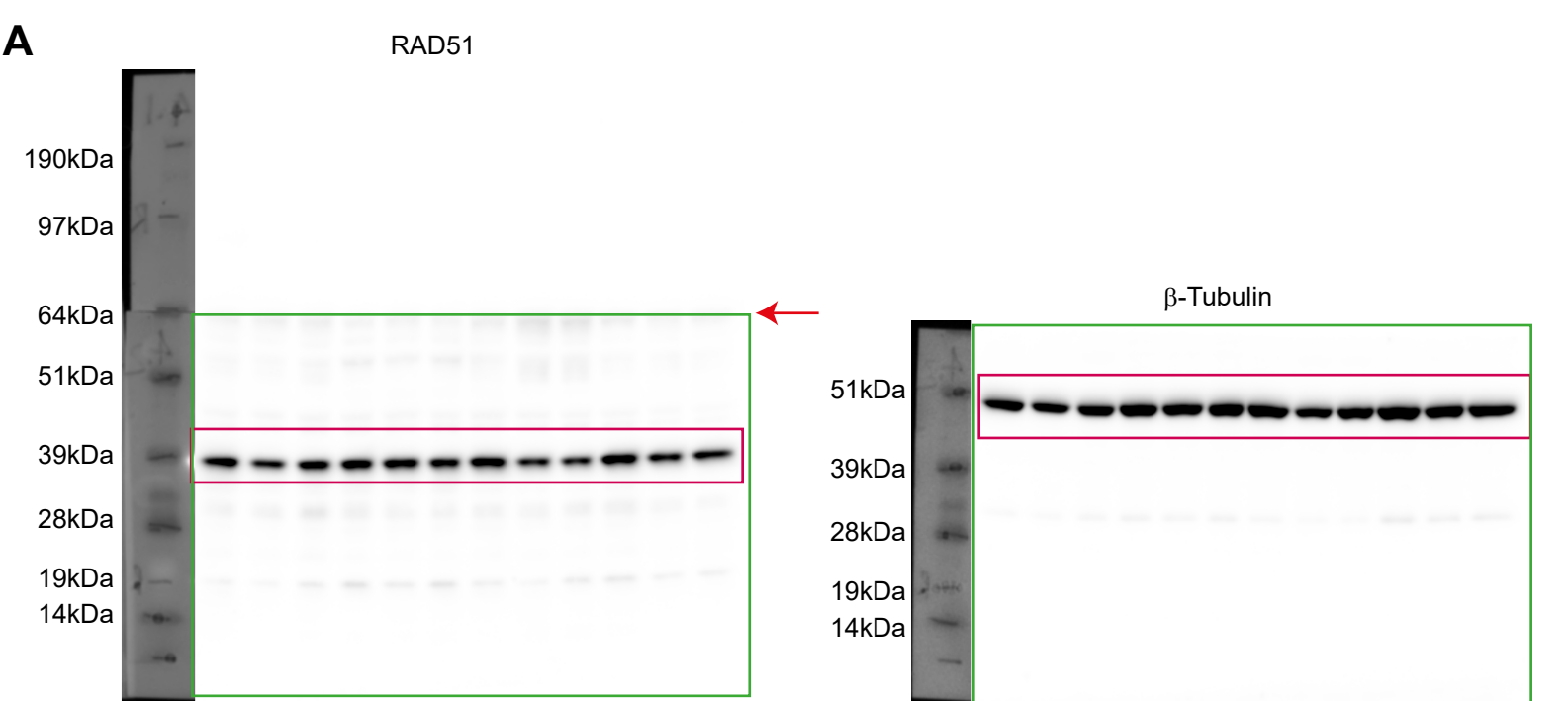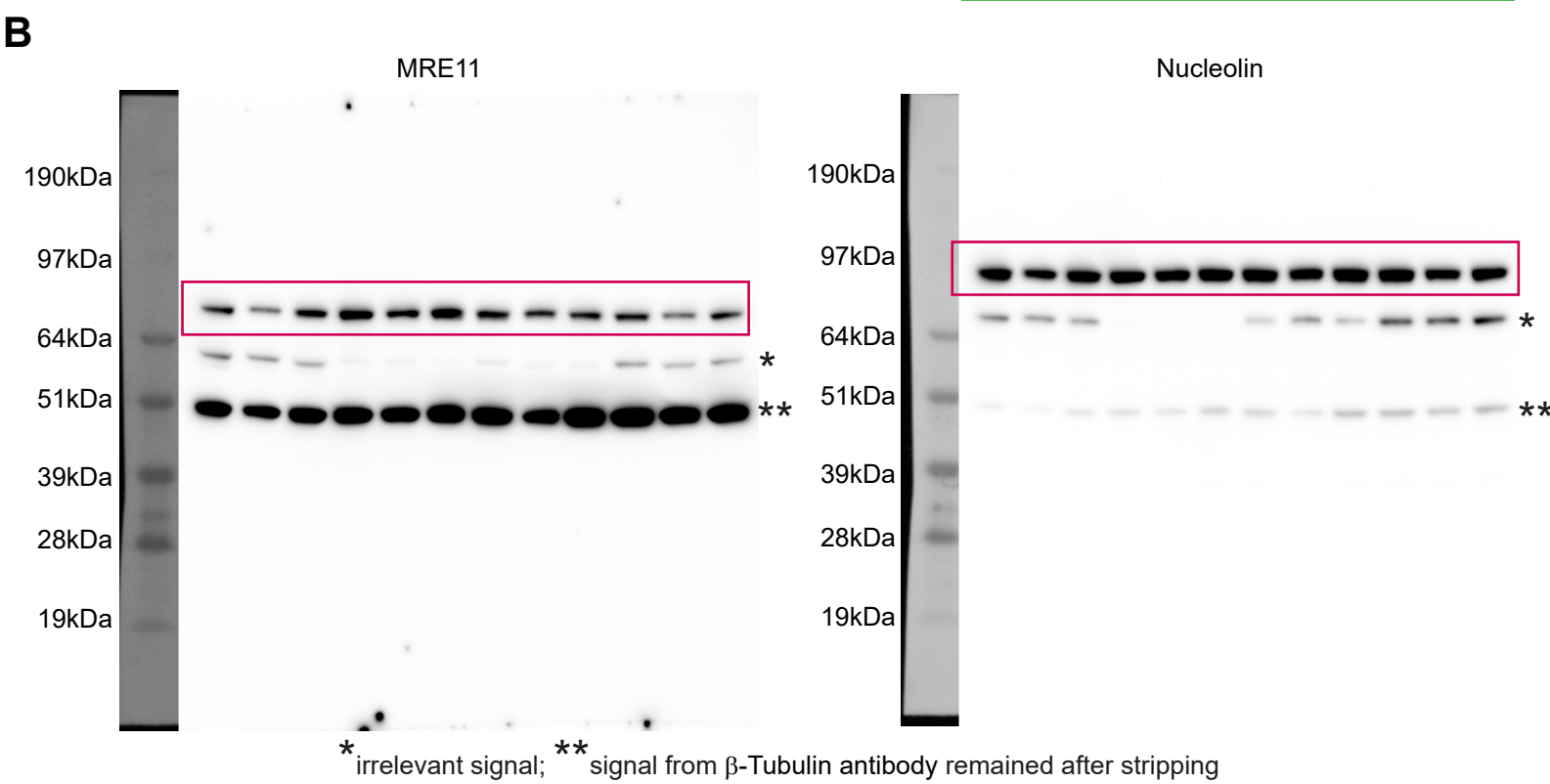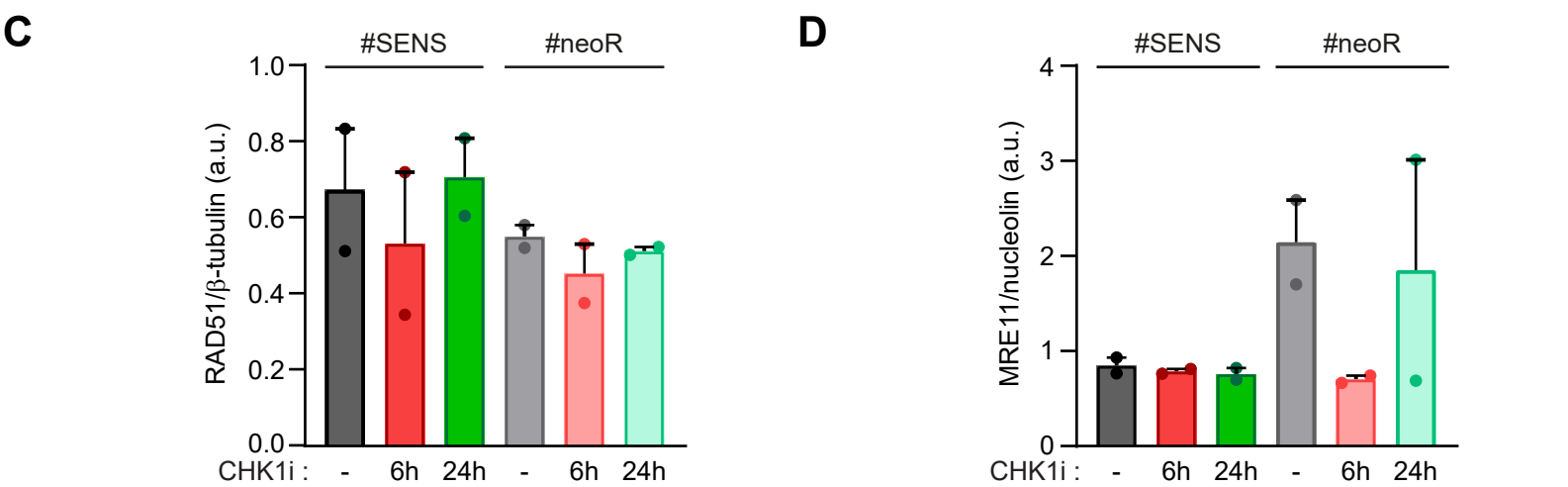

**FIGURE S2**

**A**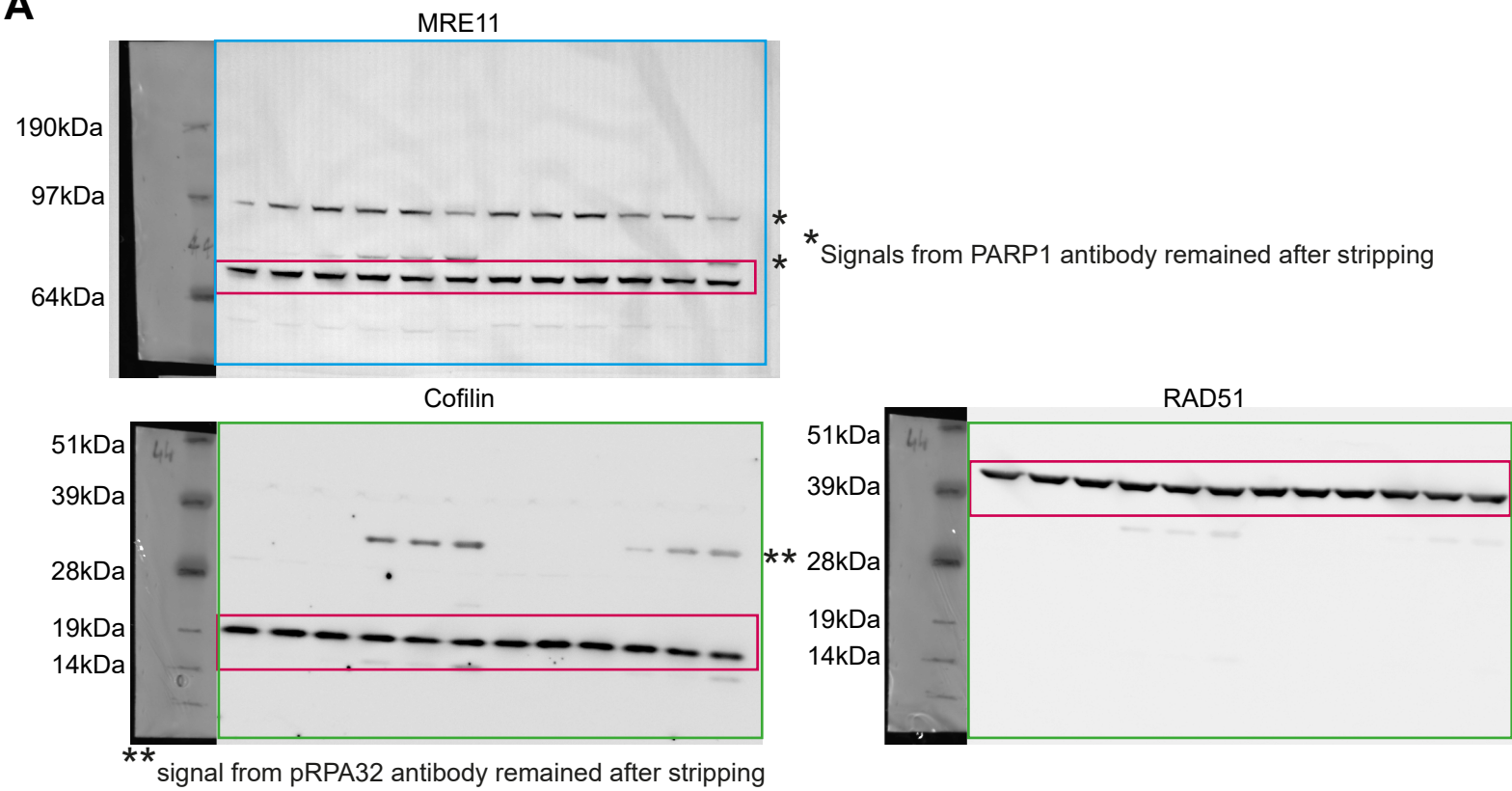**B**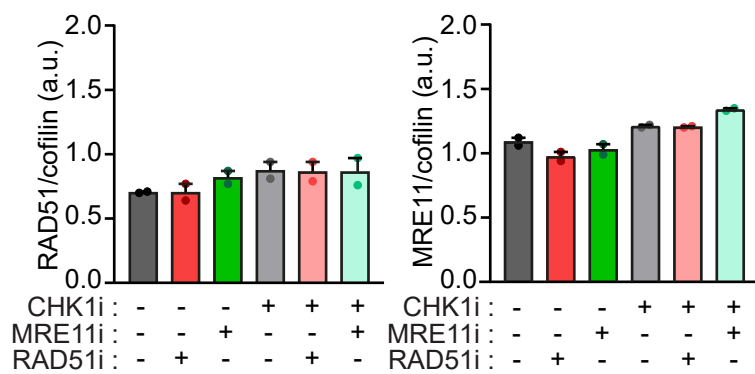**FIGURE S3**

PARP1 (up) and cPARP1 (down)

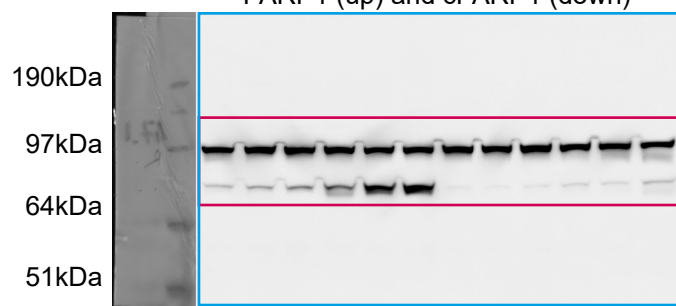

$\beta$ -Actin

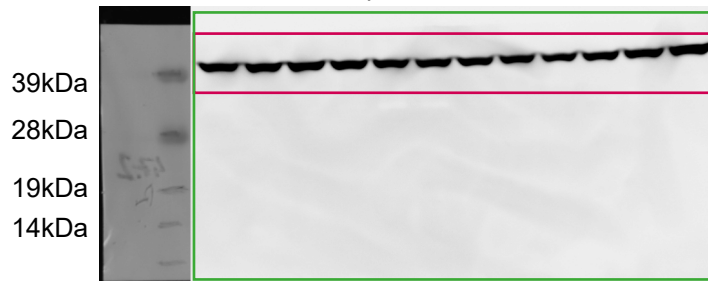

**FIGURE S4**

**A**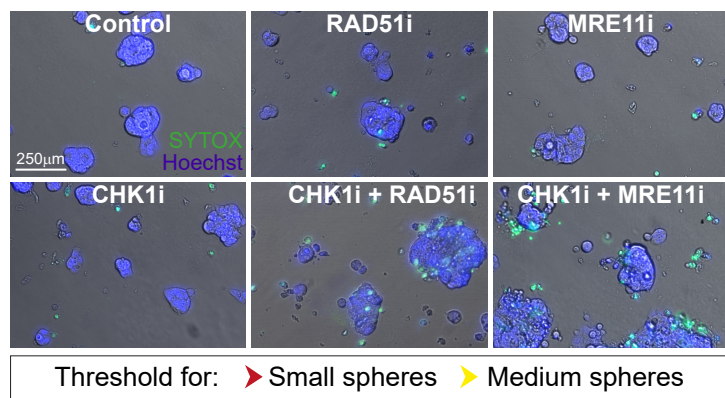**B**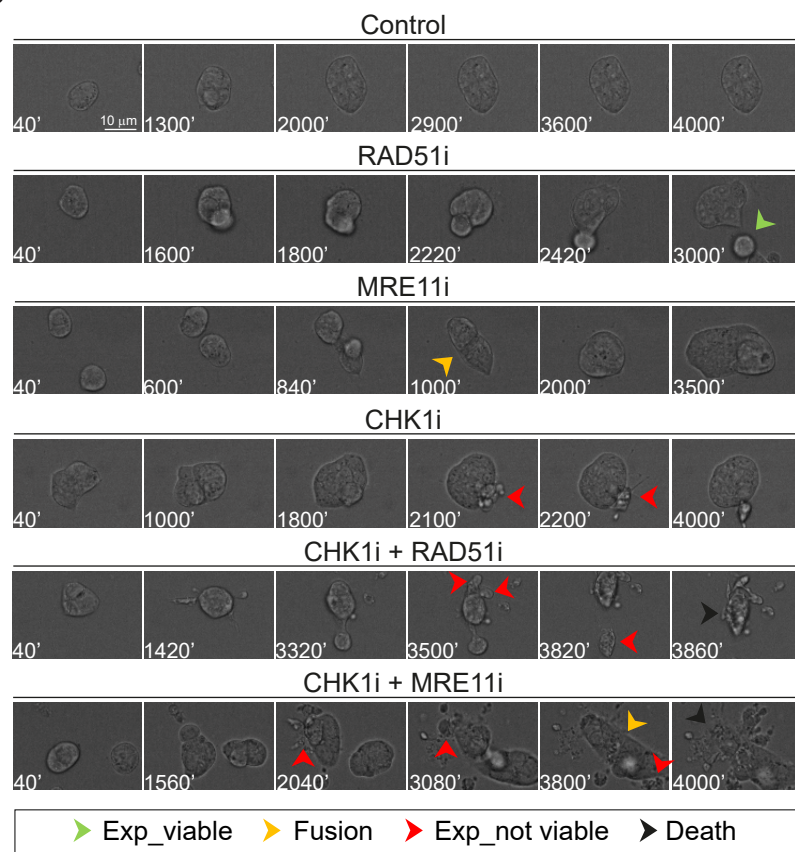**FIGURE S5**

Supplement: Supplementary file 1 [file cancers-13-01957-s001.zip › Supplementary Figures_Mattiello et al.pdf]
